# Trends in Moral Injury, Distress, and Resilience Factors among Healthcare Workers at the Beginning of the COVID-19 Pandemic

**DOI:** 10.3390/ijerph18020488

**Published:** 2021-01-09

**Authors:** Stella E. Hines, Katherine H. Chin, Danielle R. Glick, Emerson M. Wickwire

**Affiliations:** Department of Medicine, The University of Maryland School of Medicine, Baltimore, MD 21201, USA; katherine.chin@som.umaryland.edu (K.H.C.); danielle.glick@som.umaryland.edu (D.R.G.); ewickwire@som.umaryland.edu (E.M.W.)

**Keywords:** moral injury, stress, healthcare worker, COVID-19, PTSD, burnout, longitudinal, physician, resident, resilience

## Abstract

The coronavirus severe acute respiratory syndrome (COVID-19) pandemic has placed increased stress on healthcare workers (HCWs). While anxiety and post-traumatic stress have been evaluated in HCWs during previous pandemics, moral injury, a construct historically evaluated in military populations, has not. We hypothesized that the experience of moral injury and psychiatric distress among HCWs would increase over time during the pandemic and vary with resiliency factors. From a convenience sample, we performed an email-based, longitudinal survey of HCWs at a tertiary care hospital between March and July 2020. Surveys measured occupational and resilience factors and psychiatric distress and moral injury, assessed by the Impact of Events Scale-Revised and the Moral Injury Events Scale, respectively. Responses were assessed at baseline, 1-month, and 3-month time points. Moral injury remained stable over three months, while distress declined. A supportive workplace environment was related to lower moral injury whereas a stressful, less supportive environment was associated with increased moral injury. Distress was not affected by any baseline occupational or resiliency factors, though poor sleep at baseline predicted more distress. Overall, our data suggest that attention to improving workplace support and lowering workplace stress may protect HCWs from adverse emotional outcomes.

## 1. Introduction

Increased healthcare worker (HCW) distress and psychiatric morbidity have been associated with public health disasters and biological threats. Such events have included the 2003 severe acute respiratory syndrome (SARS) outbreak, the 2009 H1N1 influenza outbreak and the 2014 Ebola outbreak [1,2,3,4,5,6]. In each case, measures of HCW distress have been elevated, including symptoms of stress, anxiety, exhaustion and symptoms related to post-traumatic stress disorder. In recognition of these consequences and associated risks, the World Health Organization has recognized HCW stress as an important factor impacting patient safety and occupational health [7].

In addition to traditional domains of HCW distress, burnout is increasingly recognized as contributing to HCW psychiatric morbidity and negatively impacting job performance, quality of patient care, job satisfaction and other key metrics of HCW performance [8]. HCW burnout is a reaction to chronic stress marked by emotional exhaustion, depersonalization and low sense of personal accomplishment [9]. Recent theories, however, have proposed that HCW burnout is actually a manifestation of moral injury [10,11]. As described by Litz, moral injury reflects the psychosocial, behavioral and even spiritual impacts of “failing to prevent, or bearing witness to acts that transgress deeply held moral beliefs and expectations” [12]. Moral injury has traditionally been evaluated in the context of military service members experiencing post-traumatic stress disorder (PTSD) secondary to job duties. It thus also makes intuitive sense that moral injury might also be experienced by HCWs, particularly in provision of critical care services during periods of potentially severe stress such as during pandemic response. If HCWs experience moral injury rather than a traditional notion of burnout, long term ramifications on the health of HCWs and their organizations and implications on prevention might be quite different. For example, in a study about moral distress, defined as ethically knowing the right thing to do but being not being able to accomplish it due to institutional constraints, 15% of nurses reported having resigned a position in the past due to moral distress [13]. This example is important because it highlights that the costs of moral distress are borne not only by HCWs but also their employers through diminished employee productivity and increased turnover. When the costs of moral distress impact HCWs disproportionately and impair their abilities to achieve personal, social and economic goals, they affect social justice [14].

During the past year, the evolution of the coronavirus severe acute respiratory syndrome (COVID-19) pandemic has presented occupational and domestic stressors to HCWs. These include modifications in recommendations for use or lack of personal protective equipment (e.g., respirators), uncertainty related to transmissibility factors, delays in patient testing and diagnosis, triage of scarce resources, provision of ineffectual care and exposure to high patient morbidity and mortality, and inability to allow therapeutic family presence at the patient bedside, among others [15,16]. These events may not only be stressors, but may also induce moral injury. Although previous evaluations of HCWs exposed to stressors from pandemics and other biodisasters have studied aspects of anxiety and predictors of PTSD, none have specifically evaluated moral injury. If markers of moral injury can be identified earlier, interventions may be implemented to prevent long-term consequences such as burnout, depression or PTSD [4]. Prevention of such avoidable physical and psychological work-related injuries in all workers is a key tenet in achieving occupational justice and allowing workers to realize their full potential [14].

Factors that mitigate the consequences of mental distress following an adverse experience are termed resilience factors. These factors are often assessed at the individual, as opposed to institutional, level. Resilience factors may include having a strong sense of purpose, ability to adapt and cope, positive mental state, confidence, optimism and perceiving strong social support [17]. A 2020 systematic review evaluating psychological interventions to promote resilience in health workers found that resilience training did, indeed, improve measures of resilience and acutely reduced symptoms of depression and stress [17]. Because some resilience factors are modifiable, and interventions do show promise, it is important to identify the resilience factors that may be particularly relevant to the experience of HCWs during COVID-19 response. Intervening on these factors may alleviate long-term consequences of COVID-19-related stress on HCWs’ mental health.

The purpose of the current project was to quantify experience of moral injury and distress in HCWs during the first three months of the COVID-19 pandemic response. We previously showed that at the beginning of the US surge, HCW moral injury was similar in severity to that reported by military service members returning from combat deployments and was positively related to percentage of work in inpatient care and sleep disturbance [18]. We also saw that psychiatric distress was similar in severity to that reported by Chinese HCWs at the peak of cases in China, and was positively associated with percentage of work in inpatient care, stressful work environment, sleep disturbance and frequency of working nontraditional shifts. Based on these findings from our prior survey, we hypothesized that moral injury and distress scores would increase over the first three months of the COVID-19 pandemic and would be influenced by resiliency factors such as job type, social support and sleep disturbance.

## 2. Materials and Methods

This study was a prospective, longitudinal survey of healthcare workers performed between March and July 2020 during the first COVID-19 surge in the United States. We used a convenience sample of physicians and professional staff members included on email rosters (*n* = 838) of the Department of Medicine and the Department of Emergency Medicine as well as the Critical Care distribution list at the University of Maryland School of Medicine. The study was approved by the University of Maryland, Baltimore Human Research Protections Office (IRB HP-00090729).

We disseminated electronic surveys using the REDCap platform hosted at the University of Maryland, Baltimore [19,20]. Surveys were delivered to all recipients of the three recruitment rosters in late March 2020. Recipients received an introduction email that described the purpose of the survey and included elements of informed consent. Those wishing to complete the survey selected a link to proceed to the survey. Participants who completed the initial survey and provided an email address for subsequent correspondence received additional surveys one and three months later. Each survey was designed to take less than 5 min for completion.

Surveys collected basic demographic and occupational information and queried participants about resilience factors, distress and experience of morally injurious events. Resilience was assessed via six items written for this study and based on domains of resilience identified in prior literature: perceived workplace stress, perceived workplace support, perceived social support, positive affect experienced prior to COVID-19 (i.e., “how often did you feel positive or happy”), frequency of working nontraditional shifts and frequency of sleep disturbances [21,22]. All resilience items were scored from 1 to 5 (“not at all” to “very”), with higher scores indicating more of a given construct. Distress was evaluated using the Impact of Events Scale-Revised (IES-R) [23]. This scale has been used in prior studies to evaluate healthcare worker distress and was used to assess distress in Chinese healthcare workers in January of 2020 at the peak of the COVID-19 outbreak in China [24]. Moral injury was assessed using the Moral Injury Events Scale (MIES) [25]. This scale has previously been used to assess moral injury in military service members [25,26]. 

## 3. Analytic Plan

We performed descriptive statistics and calculated means and standard deviations (for continuous variables) and frequencies (for categorical variables) for responses measured at three months. To test the hypothesis that moral injury and psychiatric distress increased over time, we compared mean scores between baseline and one-month follow-up and separately between one-month and three-month follow-up. To test the hypothesis that predictors of distress and moral injury early in the pandemic would continue to predict persistent distress and moral injury at three months, we performed hierarchical multiple regression analyses. First, the assumptions for a linear regression were evaluated. After these were found to be tenable, two separate hierarchical multiple regression analyses were performed. First, a sequential model was developed to understand the impact of demographic (step 1), occupational (step 2), prior distress (i.e., baseline IES-R score; step 3) and resilience factors (step 4) on distress as measured via total IES-R score. Second, a similar model was developed for moral injury, with prior moral injury (i.e., baseline MIES score) being entered in step 3. For predictive analyses, sex, proportion of inpatient time and proportion of clinical time were bifurcated (male/female and >50%/<50%, respectively). Resilience items were evaluated for normality, then entered as continuous variables. Cases were excluded pairwise based on missing data. Analyses were performed using IBM SPSS v26 (IBM, Armonk, NY, USA).

## 4. Results

### 4.1. Participants

Ninety-six respondents completed the three-month follow-up survey (43.8% of *n* = 219 original respondents), 77 of whom also completed the one-month assessment. Table 1 presents demographic and occupational characteristics for these respondents. At three-month follow-up, the internal consistency reliability of the IES-R total score was α = 0.90, and internal consistency reliability of the MIES total score was α = 0.93. 

### 4.2. Trends in Moral Injury, Distress, and Resilience

Table 2 presents mean MIES, IES-R and resiliency factor scores at baseline, one-month, and three-month follow-up. Total MIES scores did not significantly change between baseline and month one or between month one and month three. Total IES-R scores significantly decreased between baseline and month one, and again between month one and month three. Among resilience factors, reported workplace stress level decreased significantly from baseline to month one. Social support increased between baseline and month one, but significantly fell between month one and month three, although it remained strong. Other factors showed no significant change. 

### 4.3. Predictors of Moral Injury and Distress at Three Months

Table 3 presents results from hierarchical multiple regression to evaluate the impact of baseline variables on three-month outcomes. In terms of moral injury, in the final model (Step 4) moral injury and stressful work environment at baseline demonstrated significant positive associations with total MIES scores at three-month follow-up. Conversely, greater supportive workplace environment at baseline was demonstrated a nearly significant (*p* = 0.08) inverse association with this dependent variable. In terms of psychiatric distress in the final model, none of the demographic, occupational or baseline distress or resilience factors were significantly associated with the dependent variable. Greater sleep disturbance at baseline showed a nearly significant (*p* = 0.09) positive association with distress score.

## 5. Discussion

In this longitudinal study of HCWs during the onset of the COVID-19 pandemic, we found that distress scores decreased between March and June 2020. However, moral injury scores remained stable. These findings were counter to our hypothesis that both outcomes would increase. None of the demographic, occupational or resilience factors assessed at baseline significantly influenced distress score at three months. Sleep disturbance showed a trend towards association, which was likely limited by statistical power. However, higher baseline moral injury, and more stressful and less supportive work environment at baseline, significantly predicted greater moral injury at three months. The survey intervals correlated with the initial spring 2020 surge in cases in Maryland (baseline), spring 2020 peak cases (one month), and decline (three months). Notably, moral injury scores during the peak case load increased slightly, yet work and social support scores did also. This may reflect occupational and social awareness of the impact of COVID-19-related care on healthcare worker wellbeing, concurrent with presence of stories in news and social media outlets.

Almost one year into the COVID-19 pandemic, many studies have examined a variety of mental health outcomes associated with pandemic-induced distress. Most of these studies have been cross-sectional, although some longitudinal studies currently exist. In comparison, our findings of decreasing distress over time are like those observed among resident physicians in Singapore [27]. There, lower stress was observed at three months, and significant predictors included living alone, less ability to problem solve, and seeking social support [27]). Our findings also echo the pattern observed in the Chinese general population, where psychiatric distress decreased significantly between the COVID-19 onset and peak number of cases one month later [28]. In that population, protective factors included high level of confidence in doctors, perceived survival likelihood, low risk of contracting COVID-19, satisfaction with health information and personal precautionary measures. In contrast, psychological distress scores worsened in Japanese HCWs assessed between mid-March and mid-May 2020, while those of the Japanese public stabilized or improved [29]. Similarly, distress did not change over time in a Spanish general population, where constant news consumption was predictive of higher distress [30].

Unlike the comparison of distress scores between our study and other research, a comparison of our MIES results with other HCW-focused research is difficult [15,31]. Therefore, we are unable to comment on how our findings of moral injury scores remaining stable might resemble findings observed in other settings. A qualitative study analyzed responses of recently deployed military nurses and physicians about traumatic exposures using a moral injury analysis framework [32]. While negative psychosocial consequences were easily identified, the authors could not assess the degree to which moral injury contributed to these outcomes. In another study of preclinical medical students regarding experience with trauma, moral injury themes resonated with interview respondents [33]. These themes included being troubled about acts they had witnessed, and being concerned about inability to act responsively [33]. 

Our findings’ importance lies in whether the reported scores can predict development of a mental health disorder such as PTSD. Several authors have proposed threshold scores for the IES-R that correlate with clinical diagnosis of PTSD, ranging from 27 to 33 [34,35,36,37]. IES-R scores in our population, even in the entire cohort (23.44 ± 13.80, *n* = 219) sampled at baseline in late March 2020, did not reach this level [18]. They were similar in scale, however, to that reported among Chinese HCWs during the peak of their reported COVID-19 cases in late January 2020 (20.0, IQR (7.0–31.0), *n* = 1257) [24]. This may provide some reassurance that HCWs were able to cope with traumatic experiences during the first months of the pandemic. 

It is unclear, however, why moral injury remained constant in this population. Total MIES scores measured in the longitudinal subgroup (range 14.36 to 15.40), and in the entire baseline cohort (16.15 ± 7.80, *n* = 219), were lower than those seen in military service members [18,25]. Subscale scores for “betrayals by others” in the initial cohort, however, (2.10 ± 1.28, *n* = 219) were similar to those reported in soldiers running from combat deployments [25]. In previous work in military populations, MIES scores, particularly in the subdomains of transgressions of others and betrayal, were most strongly associated with PTSD symptoms [26]. Whether the betrayals or transgressions of others experienced by HCWs could achieve the same impact as that experienced during combat is not known.

Studies primarily involving nurses have assessed the concept of moral distress. This is defined as knowing the right thing to do, but being unable to do it because of institutional constraints [38]. Moral distress survey results in various nursing settings showed moderately high levels even in non-pandemic times [13,39]. Moral distress has shown correlations with negative outcomes, but importantly with job abandonment and career change [13,39,40,41,42,43,44]. Job abandonment, while clearly significant for an individual HCW, also impacts an organization on multiple levels. Purely from a financial standpoint, a 2004 study showed that costs associated with turnover of physicians and registered nurses were $66,137 and $23,487 per worker [45]. These costs in 2020 values have likely increased further. Organizations have a need to prevent moral distress, if not from an ethical standpoint, then from a financial one. If moral injury and moral distress reflect the same constructs, there is clearly a need to mitigate their effects to preserve the individual and health systems alike.

From a social justice perspective, the imbalance of high COVID-19-related risks and consequences faced by HCWs compared to the general population places a disproportionate burden on this worker population. These risks are clearly present from the chance of infection and death, but also of psychological burden, overwork and time away from loved ones. HCWs have long experienced a duty to care [46]. Sacrifice of personal wants for the benefit of patients is not new. HCWs take on long work shifts, care of dying patients and difficult conversations with grieving or angry family members as a regular part of work. Ethically, however, there must be balance between duty to care and worker well-being [46]. Protection from the consequences of occupational exposure to moral injury may require a similar framework as HCW protection from blood-borne pathogen or tuberculosis exposure. Thinking of this social and occupational justice issue more broadly, just as a coal miner should be protected from the known risks of inhalation of coal mine dust, HCWs deserve protection from the consequences of moral injury. The societal benefits derived from the efforts and talents of HCWs caring for patients justify hazard reduction and mitigation of the moral toll of moral distress [43].

This study has several limitations, the first being sample size. We utilized a convenience sample of readily available email distribution lists to recruit participants. While a larger sample size was desired, one of the critical elements of this study was time-related, and we needed to capture responses as close to the onset of the initial COVID-19 surge as possible. This allowed us to measure outcomes at one month, which correlated with our peak of COVID-19 spring cases (over 34,000 cases in the state of Maryland as of 12 May 2020) [47]. Next, our population predominantly included physicians, and their responses may not be generalizable to other HCWs.

As is often seen in longitudinal studies, some participants from the baseline survey did not go on to complete the subsequent surveys. We do not know if our findings would have been different if all original participants responded at three months. Compared to the initial population, the three-month population had slightly more men (49% vs. 43%), had worked in healthcare somewhat longer (14.0 vs. 12.5 years), and included a higher proportion of attending physicians (63% vs. 47%) and fewer resident physicians (13% vs. 20%). Distribution of specialties, however, remained similar. It is possible that the participants who dropped out may have had higher or lower amounts of distress or moral injury. Because the timing of the third survey coincided with the end of the academic year, we likely lost potential resident trainee responses due to their graduation from the training program. One could argue that longer years in the profession yields greater experience, familiarity and ability to adapt, which could mitigate the experience of moral injury and distress [33]. This may also reflect survivor bias. However, in our final models, gender, years in healthcare and professional category did not significantly predict moral injury or distress scores at baseline or at three months [18]. Thus, the consistency of this finding may provide some reassurance that we did not miss a significant predictive finding because of the dropout rate. In addition, we do not know how much moral injury existed in this study population before COVID-19 onset. The MIES tool, however, asked HCW to respond considering their experiences working as HCWs in the last seven days. This discrete time interval may be well-suited to assessing moral injury that is temporally correlated with recent pandemic-related events. Thus, we believe we used a tool that could provide insight on acute, recent morally injurious experiences.

Additionally, we used a tool to quantify moral injury that has only been used previously in military populations. Although the psychometric qualities of the MIES are known in military populations, they are not known in HCW populations [25]. This tool may not measure the moral injury experienced by HCWs as reliably as it does in military service members.

Finally, we did not measure specific contributors to moral injury or distress. Recent publications have cited or speculated about the impact of numerous determinants associated with mental health outcomes during the COVID-19 pandemic. These have included having personal protective equipment and infection control needs met, fear of stigma or discrimination for working as a HCW, fear of spreading disease to a family member, domestic caregiver status, uncertainty regarding mode of disease transmission, income loss, perceived susceptibility, and vaccine availability, safety and uptake hesitancy [48,49,50,51,52,53,54,55]. These factors are all likely highly important, and we are not able to opine on which ones were most relevant in our study population.

Strengths of our study include longitudinal data, coincident with the development, peak and decline of COVID-19 case burden in Maryland. This provides crucial contextual information to help understand how mental health responses correlated with pandemic-related case activity. While many other studies have evaluated cross sectional data, only a few have published thus far on longitudinal change; none have specifically quantified moral injury. Additionally, we used the IES-R. This tool has demonstrated validity for use in COVID-19 and has been used to survey healthcare workers and the general population during COVID-19 around the world [24,30,37,56,57,58]. Thus, we believe we assessed an outcome that is important to stakeholders globally.

As of December 2020, cases of COVID-19 have surged again to record numbers, exceeding 78 million globally and 18 million US cases [47]. Unlike the three-month period evaluated in the spring of 2020, the rise in US cases starting in November has not plateaued. This diffuse disease presence may pose even greater challenges for HCWs by sheer patient volume. The experiences from the spring of 2020 will assuredly inform patient care and logistical operations of hospitals during this second wave. Knowing the burden of mental health morbidity experienced by HCWs from the first wave may help hospital leaders proactively implement strategies to improve HCW wellbeing during the second wave.

We also will continue to clarify significant predictors of moral injury and distress. Our future research work will evaluate the impact that domestic care-giver status plays on moral injury and distress outcomes in this population. Additionally, we are currently evaluating an expanded assessment of sleep disturbance and preferred self-care and resilience-related resources during the exponential COVID-19 case increase seen at the end of 2020.

## 6. Conclusions

With the variety of mental health outcomes that have been assessed in HCWs during the COVID-19 response, moral injury as a symptom, contributor, or even synonym for burnout, deserves additional study. While strategies exist to combat depression or anxiety, experience of moral injury may portend a longer-term consequence that is harder to treat. Decision makers should implement primary and secondary prevention programs to promote HCW resiliency. Such a program may help workers recognize and manage their own symptoms and limit psychiatric morbidity, especially during episodes of significant stress.

## Figures and Tables

**Table 1:** Demographic and occupational characteristics of participants completing baseline and 3 month surveys (*n* = 96).

		*n*, % or Mean (SD)	
Demographic characteristics			
Gender	Female	49	51.0%
	Male	47	49.0%
Age (years)		40.6	(10.4)
Years in healthcare		14.0	(10.3)
Professional category	Attending physician	60	62.5%
	Fellow physician	14	14.6%
	Resident physician	12	12.5%
	Other *	10	10.3%
Specialty	Hospital Medicine	12	12.6%
	PCCM	20	21.1%
	EM	18	18.9%
	All other IM (primary care and subspecialty)	36	37.9%
	Other ^†^	9	9.5%
Primary role in critical care		25	26.0%
Proportion of working time during COVID-19 response in clinical duties	<50%	42	43.8%
	>50%	54	56.3%
Proportion of working time during COVID-19 response in inpatient care	<50%	62	64.6%
	>50%	34	35.4%

* Other includes nurse practitioner or physician assistant (4), nurse (1), pharmacist (1), allied health (3), or nonclinical (1); † Other includes Anesthesia, Surgery (general & subspecialty), Neurocritical care and Not Applicable. PCCM = pulmonary & critical care medicine; EM = emergency medicine; IM = internal medicine.

**Table 2:** Moral injury, distress, and resiliency scores over 3 months during initial COVID-19 US Surge in Baltimore, MD healthcare workers (*n* = 96).

	Baseline	1 Month *	3 Month
	mean (sd)		
Moral injury (MIES)			
Total MIES ^††^	14.51	15.40	14.36
	(7.22)	(7.31)	(8.22)
Distress (IES-R)			
Total IES-R ^¶^	22.03	18.62 ^∞^	14.45 ^∞^
	(13.41)	(11.19)	(10.59)
Resiliency			
Stressful work environment ^‡^	3.34	3.04 ^∞^	3.00
	(0.96)	(0.95)	(0.88)
Supportive work environment ^‡^	3.93	4.01	3.91
	(0.90)	(0.95)	(0.89)
Social Support ^§^	4.06	4.18	4.01 ^∞^
	(0.90)	(0.91)	(0.96)
Sleep Disturbance ^|^	2.94	2.81	2.75
	(1.16)	(1.16)	(0.95)

* *n* = 77 for week four; responses were voluntary and not all participants provided responses; ^∞^ significant change from prior, *p* < 0.05. ^††^ Score range 9–54 (higher = more moral injury). ^¶^ Max score range 0–88 (higher = more distress). ^‡^ 1 = not at all, 5 = very; ^§^ 1= not very strong, 5 = very strong; ^|^ 1= never, 5= almost always.

**Table 3:** Summary of Hierarchical Regression for Variables Predicting Three Month Moral Injury Event Score (MIES (9-item)) and Impact of Events Scale—Revised (IES-R), *n* = 96.

		MIES (9-Item) Model			IES-R Model		
	Variable	B	SE B	ß	B	SE B	ß
Step 1	Constant	20.027	5.689		31.265	6.416	
	Sex	−0.127	1.957	−0.008	2.710	2.178	0.152
	Age	−0.123	0.098	−0.145	−0.109	0.114	−0.117
Step 2	Constant	16.846	9.650		27.247	11.900	
	Sex	−0.640	2.037	−0.038	2.673	2.396	0.15
	Age	0.063	0.266	0.075	0.030	0.329	0.032
	ICU	−2.444	2.093	−0.131	−0.37	2.441	−0.019
	>50% clinical time	−3.879	2.539	−0.225	−0.566	2.952	−0.031
	>50% inpatient time	−3.879	2.539	−0.225 ***	2.343	2.902	0.124
	Years in healthcare	−0.216	0.270	−0.242	−0.141	0.334	−0.143
Step 3	Constant	10.701	8.819		19.488	11.780	
	Sex	−1.176	1.842	−0.07	1.629	2.330	0.091
	Age	0.053	0.24	0.063	−0.023	0.316	−0.025
	ICU	−2.798	1.889	−0.15	0.173	2.348	0.009
	>50% clinical time	−3.214	2.296	−0.186	0.405	2.852	0.022
	>50% inpatient time	4.297	2.315	0.242 *	1.035	2.825	0.055
	Years in healthcare	−0.203	0.243	−0.228	−0.026	0.323	−0.026
	Baseline score (MIES or IES-R, respectively)	0.535	0.124	0.44 ***	0.256	0.097	0.32 **
Step 4	Constant	10.437	12.143		42.538	14.617	
	Sex	−0.765	1.942	−0.045	0.124	2.321	0.007
	Age	0.080	0.238	0.095	−0.210	0.296	−0.225
	ICU	−3.203	1.954	−0.172	1.680	2.276	0.086
	>50% clinical time	−2.997	2.280	−0.173	0.919	2.671	0.051
	>50% inpatient time	3.059	2.301	0.172	1.889	2.671	0.100
	Years in healthcare	−0.258	0.247	−0.29	0.046	0.307	0.047
	Baseline Score (MIES or IES-R, respectively)	0.425	0.132	0.349 ***	0.158	0.102	0.197
	Stressful work	1.886	0.896	0.218 **	0.761	1.110	0.086
	Supportive work	−1.893	1.080	−0.196 *	−1.386	1.231	−0.133
	Social support	−0.815	1.308	−0.087	−2.065	1.597	−0.195
	Positive affect	1.327	1.415	0.121	−1.362	1.795	−0.111
	Nontraditional shifts	0.697	0.734	0.104	0.282	0.876	0.041
	Sleep disturbance	−0.436	0.900	−0.060	1.761	1.016	0.237

IES-R: R2 = 0.048 for Step 1 (*p* = 0.181); ΔR2 = 0.015 for Step 2 (*p* = 0.903); ΔR2 = 0.090 for Step 3 (*p* = 0.011); ΔR2 = 0.213 for Step 4 (*p* = 0.007); MIES (9-item): R2 = 0.02 for Step 1 (*p* = 0.433); ΔR2 = 0.98 for Step 2 (*p* = 0.085); ΔR2 = 0.174 for Step 3 (*p* =0.000); ΔR2 = 0.10 for Step 4 (*p* =0.088); * *p* < 0.1,** *p* < 0.05, *** *p* < 0.01.

## Data Availability

The data presented in this article are available on request from the corresponding author. The data are not publicly available due to privacy.
